# Naphthyl-Substituted Ruthenium(II)–Arene Complexes: Exploring the Impact of Binding Modes on Cytotoxicity in Cancer and Normal Cell Lines

**DOI:** 10.1155/bca/5556956

**Published:** 2025-05-04

**Authors:** Serdar Batıkan Kavukcu, Hafize Seda Vatansever, Suleyman Ilhan, Hayati Türkmen

**Affiliations:** ^1^Department of Chemistry, Faculty of Science, Ege University, Izmir, Türkiye; ^2^Department of Histology and Embryology, Faculty of Medicine, Manisa Celal Bayar University, Manisa, Türkiye; ^3^DESAM Institute, Near East University, Mersin 10, Türkiye; ^4^Department of Biology, Faculty of Engineering and Natural Sciences, Manisa Celal Bayar University, Manisa, Türkiye

**Keywords:** anticancer, antineoplastic agents, ligands, Ru(II)–arene complex, ruthenium

## Abstract

This study investigated the cytotoxic properties of three naphthyl-substituted ruthenium(II)–arene complexes (**Ru1**, **Ru2**, and **Ru3**) against various cancer cell lines (MCF-7, Caco-2, and HepG2) and a healthy cell line (Vero). Herein, we report the novel synthesis and characterization of **Ru3** for the first time. The complexes were fully characterized by ^1^H, ^13^C, and 2D NMR spectroscopies, and their interactions with DNA and bovine serum albumin (BSA) were evaluated. Binding constant (Kb) determinations revealed values of 2.95 × 10^4^ M^−1^, 2.27 × 10^4^ M^−1^, and 3.70 × 10^4^ M^−1^ for **Ru1**, **Ru2**, and **Ru3** with FS-DNA, respectively, while **Ru2** exhibited a significantly higher binding constant of 0.86 × 10^5^ M^−1^ with BSA, indicating a favorable binding interaction. Molecular docking of **Ru3** was performed against BSA, EGFR wild type (EGFRWT), and mutant EGFRT790M. **Ru3** exhibited docking scores of −178.827, −204.437, and −176.946 kJ/mol with BSA, EGFRWT, and EGFRT790M, respectively. Cytotoxicity assays revealed that **Ru1-3** exhibited superior activity against MCF-7 and Caco-2 cells compared to HepG2 cells. Following a 24-h exposure, **Ru2** exhibited an IC_50_ of 1.39 μg/mL against the Caco-2 cell line. Morphological analysis suggested that all complexes induced apoptosis in cancer cells. Notably, **Ru2** demonstrated minimal activity against Vero cells, indicating selectivity. Hirshfeld surface analysis was employed to investigate intermolecular interactions within the crystal structures of the complexes, providing insights into their molecular shapes and potential for interactions with other molecules. In conclusion, this study highlights the promising potential of naphthyl-substituted ruthenium(II) complexes as anticancer agents. Their selective cytotoxicity and ability to induce apoptosis warrant further investigation for the development of novel cancer therapies.

## 1. Introduction

Cisplatin, a platinum-based coordination complex, has revolutionized the treatment landscape for various malignancies. Discovered in the 1960s [1], it established itself as a cornerstone of numerous cancer treatments due to its potent cytotoxic (cell-killing) properties. Its mechanism of action hinges on the formation of DNA adducts, which disrupt the replication and transcription processes within cancer cells, ultimately leading to cell death [2]. Cisplatin's effectiveness extends beyond its direct cytotoxicity. It also disrupts cell division by interfering with microtubule assembly, a crucial step in mitosis [3]. This multifaceted approach to cancer cell eradication has solidified cisplatin's position as a first-line therapy for a wide spectrum of cancers, including testicular, ovarian, bladder, head and neck, lung, and cervical cancers [4–6]. Furthermore, its efficacy has been demonstrated in the treatment of hematological malignancies, such as lymphomas [7].

Despite its remarkable effectiveness, cisplatin's therapeutic application is unfortunately overshadowed by a significant drawback: its dose-dependent toxicity profile. The drug can induce a spectrum of adverse effects, most notably nephrotoxicity (kidney damage), neurotoxicity (nerve damage), ototoxicity (hearing loss), and gastrointestinal toxicities (nausea, vomiting) [8]. These side effects can significantly hamper treatment regimens, necessitate dose reductions, and ultimately compromise patient quality of life. Furthermore, the development of cisplatin resistance in some cancers hinders its long-term efficacy [9]. This ongoing challenge underscores the urgent need for novel metal-based anticancer drugs with improved pharmacological profiles.

Ruthenium, a transition metal with exciting potential in the development of next-generation chemotherapeutics, has attracted the most attention. Unlike cisplatin's platinum center, ruthenium offers a unique chemical landscape that can be exploited to design drugs with distinct mechanisms of action and, potentially, reduced toxicity compared to cisplatin. Research efforts directed toward ruthenium complexes have yielded promising candidates that exhibit potent antitumor activity in preclinical models [10].

The development of targeted cancer therapies hinges on understanding the interactions between potential drugs and key cellular components [11]. DNA, the blueprint for cellular life, is a prime target for many anticancer agents. Ruthenium(II) complexes, with their diverse coordination chemistry, offer the potential to disrupt DNA function through intercalation, where the complex inserts itself between DNA base pairs, or through other interactions like hydrogen bonding and *π*-stacking [12]. These interactions can ultimately lead to the inhibition of cell proliferation and death of cancer cells. Beyond their interaction with DNA, investigating the interaction of therapeutic candidates with proteins like bovine serum albumin (BSA) is crucial [13]. BSA, the most abundant protein in blood plasma, plays a significant role in transporting molecules throughout the body. Understanding the binding between a complex and BSA provides insights into the drug's delivery, distribution, and potential toxicity. Strong binding to BSA can enhance a drug's circulation time and improve its solubility, ultimately influencing its therapeutic efficacy [14].

The relentless pursuit of novel and selective anticancer agents remains a cornerstone of cancer research [15, 16]. Current therapies often suffer from limitations such as broad cytotoxicity and debilitating side effects, highlighting the need for more targeted approaches [17]. Ruthenium(II) complexes have emerged as promising candidates in this domain due to their unique properties, including diverse coordination modes and potential for fine-tuning their biological activity [18, 19]. This study delves into the synthesis and characterization of three naphthyl-substituted ruthenium(II) complexes with varying binding modes. We further explore their cytotoxic potential against a panel of cancer cell lines (Caco-2, MCF-7, and HepG2) and a healthy cell line (Vero) to assess their selectivity toward cancer cells. By employing a combination of cell viability assays and morphological analysis, we aim to elucidate the mechanism of action and identify promising candidates for further development in targeted cancer therapy. By investigating both DNA and BSA binding activity, this study aims to identify ruthenium complexes that offer a more targeted approach to cancer treatment while minimizing adverse effects. The promising interactions observed with DNA and the favorable BSA binding characteristics suggest that these newly synthesized ruthenium complexes warrant further exploration as potential candidates for cancer therapy. The objective of this study is to investigate the anticancer potential of three ruthenium(II)–arene complexes by evaluating their cytotoxic activity against various cancer cell lines and a healthy cell line, analyzing their interactions with DNA and BSA, performing molecular docking simulations, and conducting Hirshfeld surface analysis to gain insights into their intermolecular interactions and structural properties.

## 2. Results and Discussion

### 2.1. Synthesis and Characterization

**Scheme 1:**
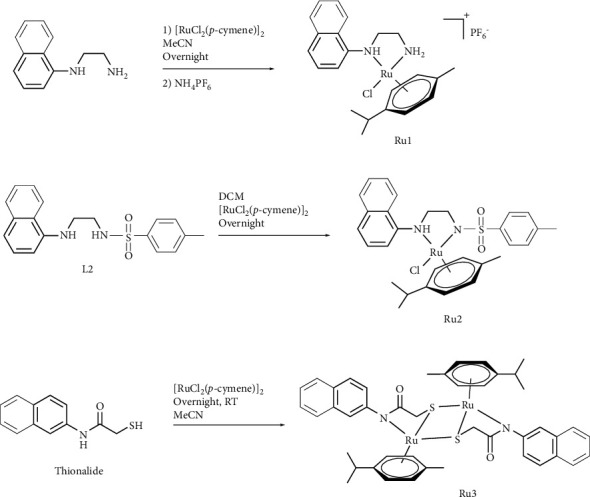
Synthesis of **Ru1–3**.

**Ru1**, the synthesis and catalytic properties of which we have previously reported, was prepared again in this study to investigate its cytotoxic properties [20].  Scheme 1 represents the synthesis of the complexes. **Ru1** was synthesized by the reaction of *N*1-(naphthalen-1-yl)ethane-1,2-diamine and (RuCl_2_[*p*-cymene])_2_. In **Ru1**, the Ru metal center bidentate bonded to the ligand with N,N donor to form a monometallic cationic complex. We reported the synthesis and catalytic properties of **Ru2** in another study, and it was synthesized again for this study [21]. **Ru2** was obtained by reaction of **L2** ligand and (RuCl_2_[*p*-cymene])_2_. **Ru2**, like **Ru1**, formed a monometallic complex by bidentate binding of the Ru metal center to the ligand with N,N donor. **Ru2** exhibited a neutral structure due to the dissociation of hydrogen bonded to the nitrogen atom close to the tosyl group. **Ru3** was obtained by reaction of commercially available thionalide and (RuCl_2_[*p*-cymene])_2_. This study details the first-time synthesis and comprehensive characterization of the novel ruthenium complex, **Ru3**. In **Ru3**, the Ru metal center is bound to the ligand as a tridentate with N,S,S donor to form a bimetallic neutral dimeric complex. In the NMR spectrum of **Ru1**, *p*-cymene aromatic protons were observed as a doublet. While two of the NH protons were observed around 6.5 ppm, one NH proton was observed at 4.90 ppm. The reason for this difference is that one of the protons with different orientations is closer to the metal center and therefore observed at lower ppm. Other aromatic and aliphatic protons show compatibility with the structure. In the NMR spectrum of **Ru2**, aromatic protons originating from the naphthyl and phenyl groups were observed in the range of 7–9 ppm, while aliphatic protons were observed in the range of 1–3.5 ppm. Arene *p*-cymene ring did not exhibit a smooth cleavage, but they were separated as one proton as in other complexes. **Ru2** was obtained as a neutral complex as a result of the breakage of hydrogen close to the tosyl group during the reaction. The NMR spectrum of **Ru3** shows splitting due to the symmetry in the structure. Aromatic *p*-cymene protons were observed as doublets, each equivalent to two protons. The aliphatic CH_2_ protons attached to the sulfur atom were observed as a doublet after the formation of the complex. ^1^H, ^13^C, and 2D analyses confirm the structures.

### 2.2. Cell Viability and Cytotoxicity

The assessment of cytotoxicity in Caco-2 (colorectal cancer), HepG2 (liver cancer), Vero (normal kidney), and MCF-7 (breast cancer) cell lines using ruthenium complexes involved the determination of cell viability and the calculation of IC_50_ values. This approach provides a quantitative measure of the cytotoxic effects of ruthenium complexes on different cancer cell lines and normal cells, offering valuable information for future studies and potential clinical applications [22].

**Figure 1:**
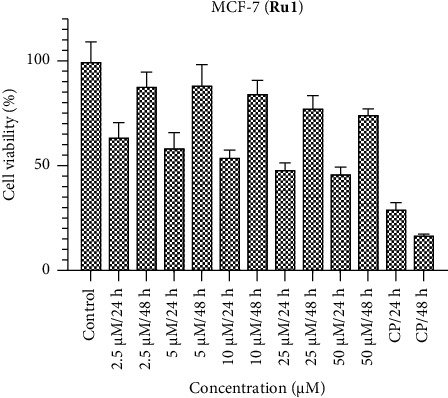
MCF-7 cell viability against five different concentrations (50–2.5 μM) of **Ru1** and 0.05 mg/mL of cisplatin in two different time intervals (24 h and 48 h).

**Figure 2:**
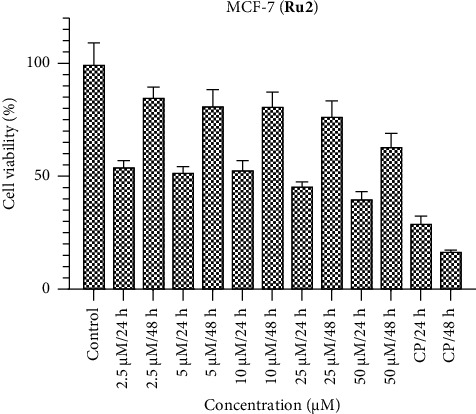
MCF-7 cell viability against five different concentrations (50–2.5 μM) of **Ru2** and 0.05 mg/mL of cisplatin in two different time intervals (24 h and 48 h).

**Figure 3:**
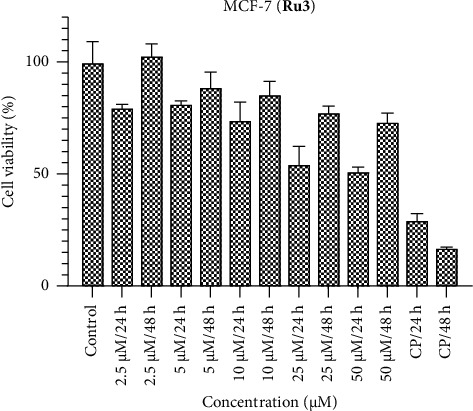
MCF-7 cell viability against five different concentrations (50–2.5 μM) of **Ru3** and 0.05 mg/mL of cisplatin in two different time intervals (24 h and 48 h).

Figures 1, 2, and 3 encapsulate an investigation into the cell viability profiles of ruthenium complexes incorporating naphthyl substituents when exposed to the MCF-7 cell line. The ensuing analyses reveal that, in terms of cell viability, **Ru2** indicates superior activity relative to its counterparts within the complex series. Furthermore, discerning variations in activity at distinct time intervals, the evaluation indicates that the efficacy of these complexes is notably more pronounced at 24 h than at 48 h. This temporal distinction suggests the potential emergence of a defense mechanism within the MCF-7 cell line against the administered complexes over prolonged exposure durations. Noteworthy parallels in activity are drawn when comparing the results with the standard chemotherapeutic agent cisplatin.

**Figure 4:**
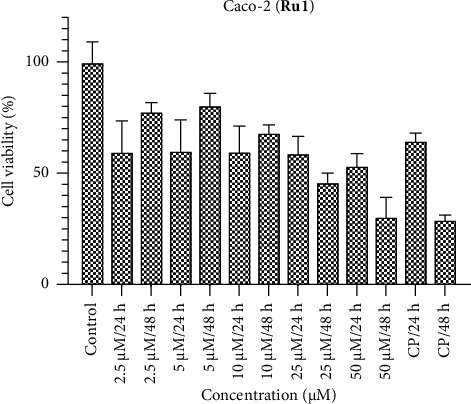
Caco-2 cell viability against five different concentrations (50–2.5 μM) of **Ru1** and 0.05 mg/mL of cisplatin in two different time intervals (24 h and 48 h).

**Figure 5:**
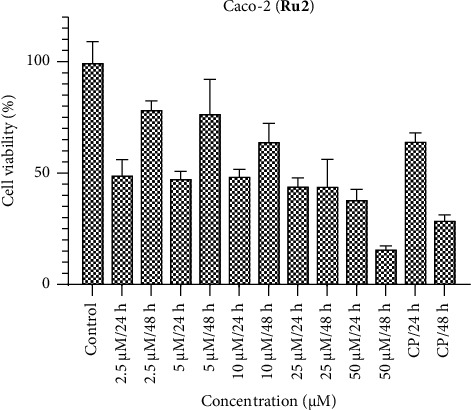
Caco-2 cell viability against five different concentrations (50–2.5 μM) of **Ru2** and 0.05 mg/mL of cisplatin in two different time intervals (24 h and 48 h).

**Figure 6:**
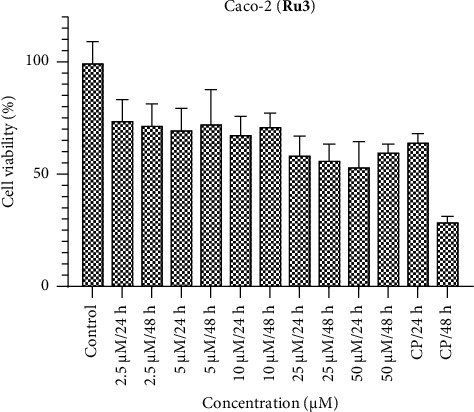
Caco-2 cell viability against five different concentrations (50–2.5 μM) of **Ru3** and 0.05 mg/mL of cisplatin in two different time intervals (24 h and 48 h).

Conversely, when Caco-2 cells are subjected to the ruthenium complexes, a discernible cytotoxic impact on cell viability is observed across all concentrations (Figures 4, 5, 6). Specifically, **Ru1** exhibits cytotoxic activity that competes with cisplatin, while **Ru2** surpasses cisplatin in efficacy according to the 24-h results. In the context of colorectal cancer cells, the **Ru3** complex, characterized by N,S,S donor ligands, displays diminished activity relative to its counterparts within the complex series. These findings contribute to a nuanced understanding of the divergent cytotoxic responses elicited by various ruthenium complexes in distinct cell lines, thereby accentuating the potential utility of certain complexes, such as **Ru2**, in anticancer applications. In **Ru1** and **Ru2** treatment, less cell viability was observed at 50 and 25 μM concentrations at 48 h, while the opposite effect was observed at other concentrations. These results show the importance of dose. It can be said that even if the defense mechanisms of cancer cells improve at high doses, the cytotoxic effect increases.

**Figure 7:**
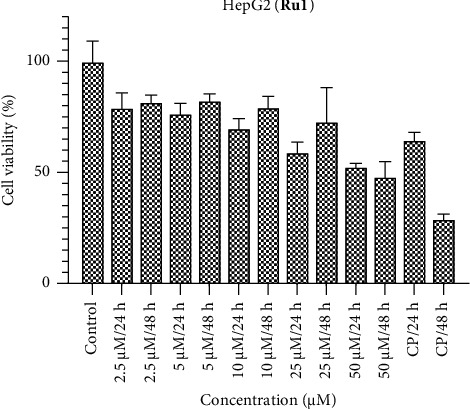
HepG2 cell viability against five different concentrations (50–2.5 μM) of **Ru1** and 0.05 mg/mL of cisplatin in two different time intervals (24 h and 48 h).

**Figure 8:**
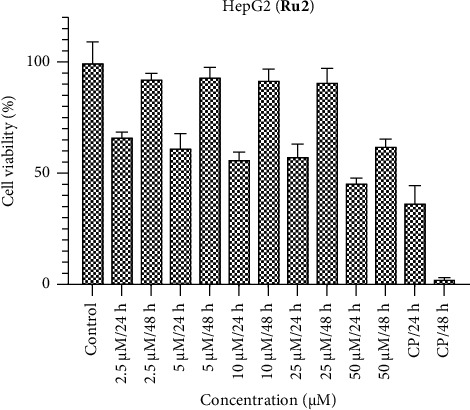
HepG2 cell viability against five different concentrations (50–2.5 μM) of **Ru2** and 0.05 mg/mL of cisplatin in two different time intervals (24 h and 48 h).

**Figure 9:**
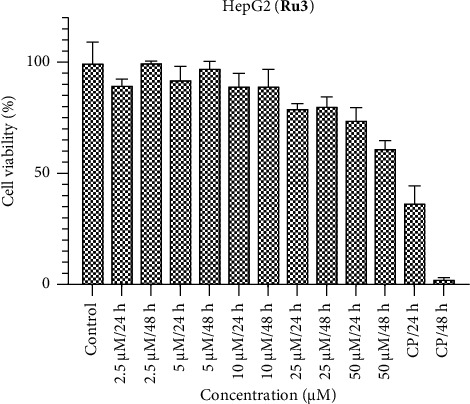
HepG2 cell viability against five different concentrations (50–2.5 μM) of **Ru3** and 0.05 mg/mL of cisplatin in two different time intervals (24 h and 48 h).

In the investigation involving the HepG2 cell line, it is notable that the observed activities of the ruthenium complexes fell below anticipated levels (Figures 7, 8, 9). In comparison with the benchmark cisplatin, the complexes displayed diminished efficacy. Particularly, the activity of **Ru2** at the 48-h interval approached a level nearly equivalent to the control, signifying a noteworthy attenuation of its cytotoxic effects. However, contrasting results were evident at the 24-h mark, where the activity of **Ru2** demonstrated a relatively competitive performance when juxtaposed with cisplatin. Conversely, **Ru3** exhibited a comparatively weakened impact in the HepG2 cell line when compared to its counterparts within the complex series. These findings underscore the nuanced and context-dependent nature of the cytotoxic responses elicited by the ruthenium complexes in HepG2 cells, underscoring the importance of temporal considerations in evaluating their antiproliferative properties.

**Figure 10:**
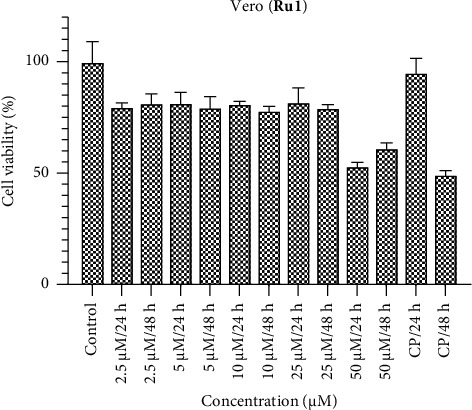
Vero cell viability against five different concentrations (50–2.5 μM) of **Ru1** and 0.05 mg/mL of cisplatin in two different time intervals (24 h and 48 h).

**Figure 11:**
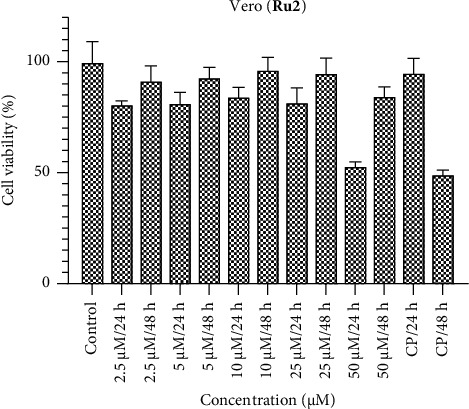
Vero cell viability against five different concentrations (50–2.5 μM) of **Ru2** and 0.05 mg/mL of cisplatin in two different time intervals (24 h and 48 h).

**Figure 12:**
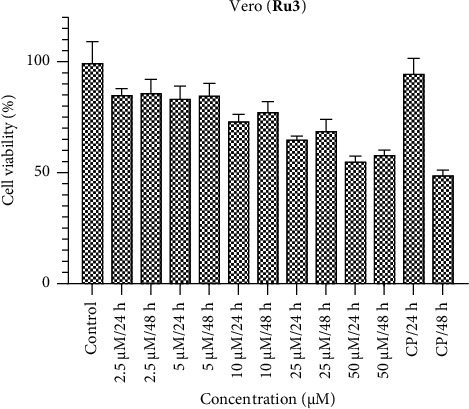
Vero cell viability against five different concentrations (50–2.5 μM) of **Ru3** and 0.05 mg/mL of cisplatin in two different time intervals (24 h and 48 h).

Within the Vero healthy cell line, it is anticipated that the ruthenium complexes would exhibit negligible cytotoxic effects (Figures 10, 11, 12). The compounds **Ru1** and **Ru2**, in particular, did not manifest any cytotoxic activity at concentrations other than 50 μM. This observation aligns with the anticipated nontoxic nature of these complexes in the Vero cell line. Conversely, the complex **Ru3** demonstrated nonselective behavior, as evidenced by its activity within healthy cells. This underlines the lack of selectivity in its cytotoxic effect, as it was observed to show activity in healthy cell apopulation. Such findings emphasize the importance of evaluating not only the cytotoxic potential but also the selectivity of Ruthenium complexes, especially in the context of their application as potential therapeutic agents. The discernment of nonselective behavior in **Ru3** underscores the need for further investigation into its cellular interactions and potential implications for therapeutic use.

The IC_50_ values of the complexes were determined following exposure durations of 24 and 48 h.  Table 1 details the time-dependent IC_50_ values for each complex across various cell lines. In the MCF-7 cell line, lower molar concentrations were observed for IC_50_ values at 24 h compared to 48 h. The most effective complex was **Ru2**, exhibiting an IC_50_ value of 2.81 μM. Conversely, the Caco-2 cell line displayed superior responses to **Ru1** and **Ru3** complexes at 48 h, with IC_50_ values of 3.83 and 1.81 μM, respectively. However, **Ru2** demonstrated greater activity at 24 h in this cell line, achieving an IC_50_ value of 1.39 μM. Notably, the significant decrease in the efficacy of **Ru2** in Vero cells (noncancerous) at 48 h compared to 24 h (38.34 μM vs. 7.80 μM) suggests a potential increase in selectivity toward cancer cells over time. The HepG2 cell line displayed no significant difference in response to **Ru1** and **Ru3** complexes between 24 and 48 h, while **Ru2** again exhibited superior activity at 24 h. These findings underscore the critical role of both dose and exposure time in determining drug efficacy, as the optimal combination can vary depending on the specific cell line.

The effectiveness of Ruthenium (Ru) complexes against various cancer cell lines (MCF-7, Caco-2, and HepG2) and a noncancerous cell line (Vero) at different exposure times (24 and 48 h) were investigated. The effectiveness was measured by IC_50_ values, which represent the concentration needed for 50% inhibition of cell growth. The IC_50_ values varied depending on the exposure time. MCF-7 cells showed better response at 24 h for all complexes, suggesting faster drug action. Caco-2 cells responded better to **Ru1** and **Ru3** at 48 h, while **Ru2** performed better at 24 h. HepG2 cells showed no significant difference between 24 and 48 h for **Ru1** and **Ru3**. Our study suggests some degree of selectivity for the complexes toward specific cell lines. For example, **Ru2** performed well in MCF-7 and Caco-2 at 24 h, while **Ru1** and **Ru3** excelled in Caco-2 at 48 h. The significant decrease in **Ru2**'s effectiveness toward Vero cells (noncancerous) at 48 h compared to 24 h might indicate increased selectivity for cancer cells over time. However, further investigation is needed to confirm this. The study highlights the importance of considering both dose and exposure time when evaluating drug efficacy. The optimal combination might vary depending on the cell line and the desired outcome. Overall, this study provides promising initial data on the potential of Ru complexes as anticancer agents. However, further research is needed to fully understand their efficacy, selectivity, and mechanism of action.

Vyas et al. investigated the cytotoxic and apoptotic properties of tetrazolate Ru(II)–arene complexes in MCF-7 and HepG2 cancer cell lines [23]. While one of the two complexes did not exhibit significant cytotoxic activity even at high micromolar concentrations, the other complex reduced cell viability to below 60% at 10 μM and higher concentrations. Wang et al. achieved an IC_50_ value of 2.63 μM in the MCF-7 cell line with their tetranuclear Ru(II)–*p*-cymene complex [24]. Rahman et al. reported a cell viability value below 30% at 20 μM concentration in the MCF-7 cancer cell line with their N,O donor bimetallic Ru(II)–*p*-cymene complex [25]. Gichumbi et al. examined the cytotoxic properties of Ru(II)–*p*-cymene complexes containing N,N bidentate ligands in HepG2, MCF-7, and Caco-2 cancer cell lines [26]. While the complexes exhibited IC_50_ values above 100 μM in Caco-2 and HepG2 cell lines, they achieved a maximum IC_50_ value of 46.6 μM in the MCF-7 cell line.

The Ruthenium(II)–arene complexes synthesized in this study have exhibited remarkable cytotoxic activity compared to structures reported in the literature. The high efficacy observed, particularly against MCF-7 and Caco-2 cell lines, demonstrates that these complexes are more successful in targeting cancer cells than their counterparts [27, 28]. The IC_50_ values in the low nanomolar range suggest that the complexes possess potent cytotoxic effects that could potentially be used in clinical applications. Furthermore, the selectivity property, which is not observed in most studies in the literature, that is, low toxicity against normal cells, further enhances the therapeutic potential of these complexes. These results support that the synthesized ruthenium(II)–arene complexes are more promising anticancer agents than similar structures in the literature.

### 2.3. Cell Morphology

The changes in cell morphology of **Ru1**, **Ru2**, and **Ru3** complexes in Caco-2 (colorectal cancer), HepG2 (liver cancer), Vero (normal kidney), and MCF-7 (breast cancer) cell lines after 24 h and 25 μM treatment were investigated. All images are available in supporting information. This study was conducted to understand factors such as cell health, growth rate, apoptosis (programmed cell death), and other cellular events. Cell morphological changes include changes in the shape, size, and structural properties of cells. These changes are used to evaluate the effects of substances or compounds on cells.

Following treatment, a statistically significant reduction in MCF-7 cell viability was observed, with **Ru2** exhibiting a particularly pronounced effect as evidenced by cellular imaging. Upon comprehensive evaluation of all complexes, discernible alterations in cellular morphology provide compelling evidence for the induction of apoptotic mechanisms in cancer cells. Microscopic analysis following complex application revealed a disruption of Caco-2 cell structural integrity, characterized by cellular swelling and subsequent degenerative changes indicative of cellular disintegration. Consistent with observations in MCF-7 cells, the **Ru2** complex induced a reduction in cell population within the HepG2 cell line. The enhanced activity of the **Ru2** complex across both cell lines is supported by corresponding cell viability assays. Given that Vero cells represent a healthy cell line, minimal activity from the complexes was anticipated. The observed diminished activity of the **Ru2** complex is noteworthy, potentially suggesting selectivity. This hypothesis warrants further investigation through comprehensive studies. In summary, the comprehensive evaluation of all complexes revealed a consistent and robust induction of apoptotic pathways, thereby highlighting their promising potential as agents capable of triggering programmed cell death.

The development of novel and effective cancer therapies remains an urgent medical need [29]. Current treatments often have limitations, including lack of selectivity toward cancer cells, severe side effects, and emergence of drug resistance [30]. This study explores the potential of ruthenium(II) complexes containing naphthyl substituents as a new class of anticancer agents. By investigating their cytotoxicity against various cancer cell lines and a healthy cell line, this research aims to identify compounds that selectively target and eliminate cancer cells. The unique properties of ruthenium, coupled with the strategic design of the complexes, hold promise for overcoming some of the challenges associated with existing cancer therapies.

Our group has carried out a series of studies on the antitumor properties of Ru(II) and Ir(III)–arene complexes. In our published work, we investigated the cytotoxic properties of monometallic and bimetallic Ru(II)–*p*-cymene complexes with N,N donors in HeLa, MDA-MB-231, DU-145, LNCaP, Hep-G2, Saos-2, PC-3, and MCF-7 cancer cells and 3T3-L1 and Vero healthy cells [31]. The results highlighted the importance of metal number and ligand structure. In our next study, we synthesized a highly oil-soluble Ru(II)–*p*-cymene complex with aliphatic chain group, a bimetallic Ru(II)–*p*-cymene complex with N,S,S triple coordination, and a bimetallic Ir(III)–pentamethylcyclopentadienyl complex with S,S double coordination and investigated the cell death mechanisms in HepG2 and Vero cell lines by immunohistochemical analyses [32]. It was determined that different ligand structures and metal activated different cell death mechanisms. In another study, we investigated the cytotoxic activity of ruthenium(II) complexes exhibiting coordination with nitrogen (amine and amide), oxygen, and sulfur donor atoms coupled with aryl and aliphatic fins on MCF-7 cell line [33]. In this study, we investigated the cytotoxic properties of three different Ru(II)–*p*-cymene complexes on Caco-2, HepG2, MCF-7, and Vero cell lines and compared them with previous studies. This study investigates the cytotoxic properties of **Ru1** and **Ru2** complexes for the first time, although their catalytic properties were previously reported by our group. The **Ru3** compound, synthesized for this purpose, is also reported here for the first time.

Sadler et al. extensively explored the cytotoxic and DNA-binding properties of Ru(II)–arene complexes containing an ethylenediamine structure in human ovarian cancer cells [34]. Their work investigated how different arene groups influence the activity of these ethylenediamine-based complexes. They elucidated the crucial roles played by amine groups (NH or NH_2_), the hydrophobic arene moiety, and the chloride leaving group in the novel recognition mechanism these Ru–arene complexes exhibit toward nucleic acids. These findings hold significant promise for the development of more effective anticancer drugs and the design of novel, site-specific DNA-binding reagents [35, 36]. Other studies by Sadler and his research group have shown that the interactions of ethylenediamine Ru(II)–arene complexes with DNA are strong and DNA is the main target of these complexes [37, 38]. Furthermore, Thomet et al. investigated the cytotoxic activity of Ru anethole complexes containing ethylenediamine in HT-29 and CCD-841 cell lines. Their research suggests these complexes may be promising candidates for further drug discovery efforts [39, 40]. Our work expands upon the existing literature by exploring the activity of these complexes against a broader range of cancer cell lines and by investigating their interactions with DNA and BSA, providing valuable insights into their potential for in vivo applications.

### 2.4. DNA-Binding Activity

Ru(II)–arene complexes interact with DNA through multiple mechanisms, potentially disrupting cellular functions and leading to cancer cell death [41]. These interactions include the arene ring inserting itself between DNA base pairs (intercalation), electronic interactions between the complex's aromatic ring and DNA bases (π-stacking), hydrogen bonding between the complex and DNA, and in some cases, direct covalent bond formation between the ruthenium center and a DNA base. The strength and type of interaction depend on the size and functional groups of the arene complex, highlighting the ongoing research to optimize these molecules for targeted cancer treatment.

To investigate these interactions, UV-Vis analyses were performed on complexes **Ru1-3** (0–15 μM) in 20 mM Tris-HCl/NaCl buffer (pH 7.2) containing 0.1 mM FS-DNA. The corresponding absorption spectra are provided in the supporting information. Aromatic naphthyl groups are known for their ability to intercalate between DNA base pairs. Therefore, the synthesized complexes were anticipated to exhibit favorable interactions with DNA. Due to its higher aromatic group density, **Ru3** demonstrated the strongest interaction. Interestingly, the interaction with **Ru2** intensified at higher concentrations. While **Ru1** is a cationic complex with a high capacity for hydrogen bonding, potentially facilitating ionic interactions with DNA, its overall interaction was weaker compared to the other complexes. In conclusion, these findings suggest that DNA is a primary target for these complexes, and they display promising interactions with DNA molecules. The binding constants (*K*_*b*_) were determined to be 2.95 × 10^4^ M^−1^, 2.27 × 10^4^ M^−1^, and 3.70 × 10^4^ M^−1^ for **Ru1**, **Ru2**, and **Ru3**, respectively. Among these complexes, **Ru3** exhibited the highest binding affinity toward DNA, as evidenced by its highest binding constant.

### 2.5. BSA Binding Activity

Studying the interaction between Ru(II)–arene complexes and BSA, the major protein-transporting molecules in blood, is crucial [42]. This helps us understand how these complexes will be delivered throughout the body and potentially influence their effectiveness as drugs. The complexes primarily interact with BSA through weak, noncovalent forces like hydrogen bonding and van der Waals interactions. The structure of the complex and the protein's conformation can affect binding strength, which in turn impacts the distribution, metabolism, and potential toxicity of the Ru(II)–arene complexes within the body.

To elucidate the interaction between BSA and **Ru3**, UV-Vis spectroscopy was employed. The experiments were conducted in a 20 mM Tris-HCl/NaCl buffer solution (pH 7.2) supplemented with 0.1 mM BSA and the **Ru3** concentration was 0–15 μM. The analysis revealed a concentration-dependent enhancement in complex binding to BSA, signifying a strong affinity between the two molecules. The binding constant (*K*_*b*_) of the interaction was determined to be 0.86 × 10^5^ M^−1^, indicating a favorable binding process.

Understanding the interactions of ruthenium complexes with BSA is crucial for several reasons. BSA is the most abundant protein in blood plasma, and its binding properties can significantly influence the in vivo behavior of therapeutic agents. A strong complex–BSA interaction can enhance the drug's circulation time, improve solubility, and potentially promote targeted delivery. Therefore, this study provides valuable insights into the potential of ruthenium complexes for the development of novel therapeutic strategies.

### 2.6. Molecular Docking

The molecular docking analysis of the synthesized compound **Ru3** and the reference drug Erlotinib (EB) was conducted with three target proteins: BSA, EGFR wild type (EGFRWT), and the mutant form EGFR T790M (Table 2). The interactions of **Ru3** with BSA, EGFRWT, and EGFR T790M were analyzed to obtain a comprehensive understanding of its pharmacokinetic properties (via BSA binding) and therapeutic potential (via interactions with EGFRWT and EGFR T790M) against both sensitive and drug-resistant cancer types. This multifaceted approach will help to evaluate the efficacy and applicability of **Ru3** as a novel anticancer agent.

The docking score of **Ru3** with BSA was found to be −178.827 kcal/mol, which is lower than that of EB (−153.346 kcal/mol), indicating a stronger binding affinity of **Ru3**. Notably, **Ru3** exhibited hydrogen bond interaction energy of −3.110 kcal/mol, while EB showed a higher hydrogen bond interaction energy of −13.107 kcal/mol. The binding interactions of **Ru3** involved several amino acid residues, including ASP20, GLY17, GLY99, and ARG196, among others, indicating multiple stabilizing interactions. In contrast, EB's interactions were more diverse, involving residues such as ARG42, THR148, and LEU100.

These results suggest that **Ru3** forms a stable complex with BSA, potentially indicating better serum binding characteristics compared to EB. Enhanced binding to serum proteins like BSA could impact the pharmacokinetic profile of **Ru3**, affecting its bioavailability and distribution in vivo. However, it is also critical to consider that strong binding to BSA may reduce the free, active concentration of the drug in circulation, potentially limiting its therapeutic effects.

**Ru3** exhibited a significantly lower docking score (−204.437 kcal/mol) with EGFRWT compared to EB (−179.941 kcal/mol), suggesting a higher binding affinity. **Ru3** engaged in interactions with critical amino acid residues such as HIS846, VAL852, and PHE699, which are known to be involved in ligand stabilization within the EGFR binding pocket. Interestingly, no hydrogen bond interaction was observed for **Ru3**, unlike EB, which displayed hydrogen bond interaction energy of −4.057 kcal/mol with residues like PHE699 and ALA835.

The lack of hydrogen bonding in **Ru3**'s interaction might be compensated by strong hydrophobic and van der Waals interactions, contributing to its overall binding stability. This finding could imply that **Ru3** might interact differently with the EGFRWT binding site compared to EB, possibly offering a new inhibitory mechanism. This variation in binding interactions could potentially enhance the efficacy of **Ru3** against EGFR-driven cancers, especially in cases where resistance to EB is observed.

The docking score of **Ru3** with the mutant EGFR T790M was −176.946 kcal/mol, which is lower than that of EB (−150.700 kcal/mol), indicating a higher affinity of Ru3 for this mutant form. The interaction profile of **Ru3** included residues such as PHE856, GLU758, and LYS754, while EB primarily interacted with MET790 and MET766. Notably, **Ru3** did not show any hydrogen bond interactions, whereas EB exhibited a minor hydrogen bond interaction energy of −0.598 kcal/mol.

The stronger binding affinity of **Ru3** to the EGFRT790M mutant is particularly significant, as this mutation is a known mechanism of resistance to first-generation EGFR inhibitors like EB. The interaction of **Ru3** with diverse residues outside the typical binding region of EB suggests that **Ru3** may overcome resistance mechanisms by binding to alternative sites or forming more stable interactions within the mutant EGFR pocket.

Overall, **Ru3** demonstrated higher docking scores (indicating stronger binding affinities) compared to EB across all tested proteins, including BSA, EGFRWT, and EGFRT790M. These results highlight the potential of **Ru3** as a potent inhibitor, particularly against EGFR mutations associated with drug resistance. The diverse interaction profiles and the absence of hydrogen bonding in some cases may indicate a different mode of binding, which could be advantageous in targeting resistant cancer cells. Further experimental validation through in vitro and in vivo studies is necessary to confirm these findings and to explore the therapeutic potential of **Ru3** in overcoming resistance to current EGFR inhibitors.

### 2.7. Hirshfeld Surface Analysis

The crystal data of **Ru1** and **Ru2** complexes, whose crystal structures were obtained in our previous studies, were evaluated by Hirshfeld surface analysis in this study [20, 21]. Hirshfeld surface analysis provides valuable insights into intermolecular interactions within crystals, which can be crucial for understanding the behavior of molecules in biological systems [43]. By analyzing the nature and strength of these interactions, we can gain insights into factors like drug-receptor binding, protein–protein interactions, and crystal packing in pharmaceuticals [44]. This information can aid in the design and optimization of molecules with improved biological activity and bioavailability. The dnorm surface maps the difference between the van der Waals radii of two interacting atoms. It provides a visual representation of the deviations from the expected van der Waals contacts. Shape index surface map refers to the capacity of the molecule to perform *π*-π stacking.

**Figure 16:**
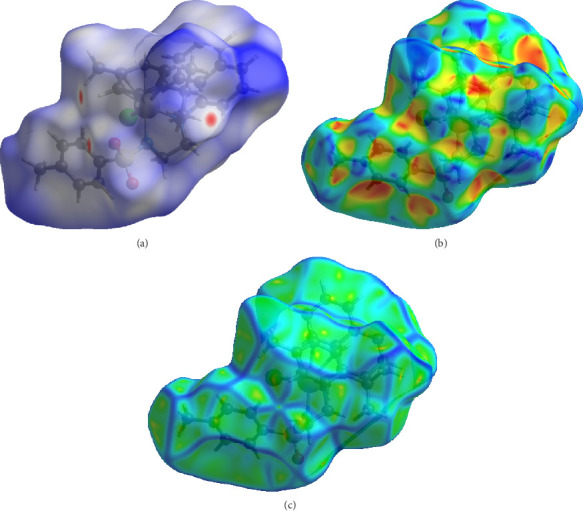
The three-dimensional Hirshfeld surface of **Ru2** plotted over dnorm (a) in the range of −0.1488 to 1.7704 a.u., plotted over shape index (b) and curvedness (c).

Figure 16 shows the dnorm, shape index, and curvedness results of **Ru2**. The dnorm image of **Ru2** reveals a significant contribution of van der Waals interactions to the crystal packing. Red regions indicate closer contacts, while blue regions indicate more distant contacts. White areas represent contacts with atoms at van der Waals distance, indicating typical van der Waals interactions. Shape index analysis of **Ru2** suggests potential sites for *π*-π stacking interactions with other molecules. The red and blue triangles indicate regions of *π*-π stacking. The curvature map of **Ru2** shows a predominantly convex surface with some concave regions, suggesting the presence of both protruding and recessed areas on the molecule's surface. These features influence the molecule's interactions with other molecules and its behavior in biological systems.

**Figure 17:**
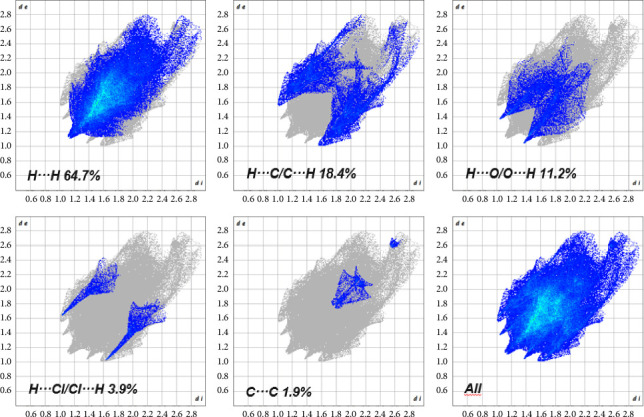
2D fingerprint plots of **Ru2** showing the contributing interactions in the total Hirshfeld field.

The 2D fingerprint plot analysis provides valuable insights into the nature and frequency of intermolecular contacts within the crystal structure of **Ru2** (Figure 17). The plot reveals that hydrogen–hydrogen interactions (H…H) are the most dominant, contributing to 64.7% of all contacts. This suggests a significant role for hydrogen bonding in stabilizing the crystal packing. Interactions between hydrogen and carbon atoms (H…C/C…H) contribute 18.4%, indicating the presence of significant van der Waals forces, likely involving aromatic rings and alkyl groups. Hydrogen bonding interactions involving oxygen atoms (H…O/O…H) contribute 11.2% to the overall interactions. Contributions from H…Cl/Cl…H and C…C interactions are relatively minor, at 3.9% and 1.9%, respectively. Collectively, these results indicate that a combination of hydrogen bonding and van der Waals forces are crucial for the stabilization of the crystal structure of **Ru2**.

**Figure 18:**
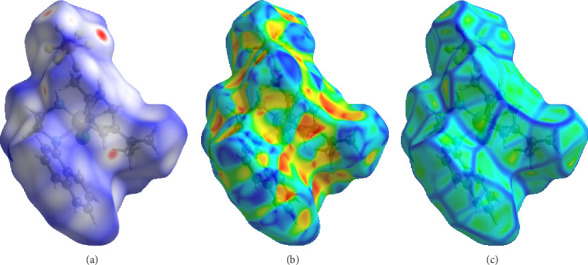
The three-dimensional Hirshfeld surface of **Ru1** plotted over dnorm (a) in the range of −0.2339 to 1.4759 a.u., plotted over shape index (b) and curvedness (c).

Figure 18 shows the dnorm, shape index, and curvedness results of **Ru1**. In the dnorm image of **Ru1**, the presence of prominent red regions indicates significant contributions of van der Waals interactions. These regions correspond to areas where the molecule is in close contact with atoms from neighboring molecules. In addition, the presence of white regions indicates significant van der Waals interactions. These interactions contribute to the overall stability of the crystal packing in **Ru1**. In shape index analysis of **Ru1**, similar to **Ru2**, blue and red triangles represent *π*-π stacking. The curvature map of **Ru1** reveals a complex surface topography with a dynamic interplay of concave and convex regions.

**Figure 19:**
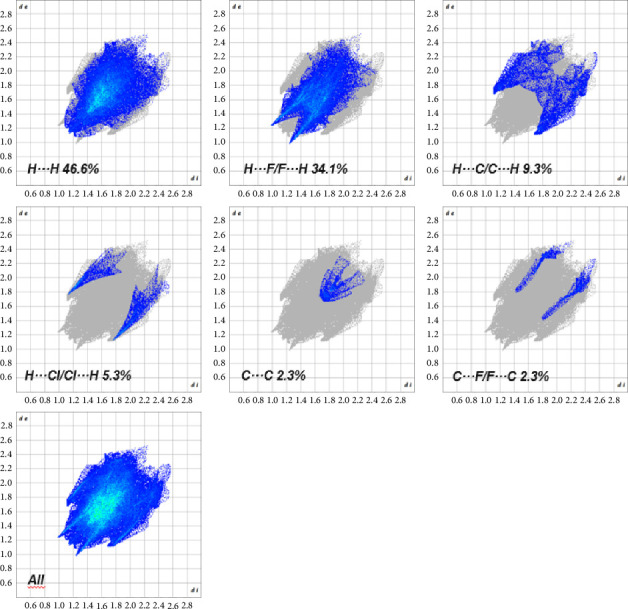
2D fingerprint plots of **Ru1** showing the contributing interactions in the total Hirshfeld field.

The 2D fingerprint plot of **Ru1** reveals that hydrogen–hydrogen interactions (H…H) are the most dominant, contributing to 46.6% of all contacts (Figure 19). This suggests a significant role for hydrogen bonding in stabilizing the crystal packing. Interestingly, interactions between hydrogen and fluorine atoms (H…F/F…H) contribute substantially to 34.1%, indicating the presence of significant fluorine–hydrogen interactions. These interactions could involve hydrogen bonds between hydrogen atoms on the Ru1 complex and fluorine atoms from PF_6_ molecules. Interactions between hydrogen and carbon atoms (H…C/C…H) contribute 9.3%, suggesting the presence of van der Waals forces, likely involving aromatic rings and alkyl groups. Contributions from H…Cl/Cl…H and C…C interactions are relatively minor, at 5.3% and 2.3%, respectively. Overall, the 2D fingerprint plot analysis reveals a diverse range of intermolecular contacts contributing to the crystal packing of **Ru1**. Hydrogen bonding interactions, both H…H and those involving fluorine atoms, appear to play a crucial role. The presence of significant H…C/C…H interactions suggests that van der Waals forces also contribute significantly to the stability of the crystal structure. This information provides valuable insights into the intermolecular forces that govern the crystal packing of **Ru1** and can be used to understand its physical and chemical properties, including its solubility and stability.

Both Hirshfeld surface analysis and 2D fingerprint plots provide valuable insights into the intermolecular interactions governing the crystal packing of **Ru1** and **Ru2**. The 2D fingerprint plots for **Ru1** reveal a significant contribution of hydrogen bonding interactions, particularly those involving hydrogen and fluorine atoms, alongside van der Waals forces. The Hirshfeld surface analysis of **Ru2**, on the other hand, indicates a dominant role of van der Waals interactions. These findings, coupled with the observed cytotoxic activities in various cancer cell lines and the results from DNA and BSA binding studies, provide a comprehensive understanding of the intermolecular interactions that govern crystal packing and potentially influence the biological behavior of these ruthenium complexes. The insights gained from these analyses can be utilized to guide future modifications to the molecular structure, aiming to optimize properties such as solubility, bioavailability, and ultimately, the anticancer activity of these promising compounds.

## 3. Conclusion

This study investigated the anticancer potential of three naphthyl-substituted ruthenium(II) complexes with varying binding modes (bidentate and tridentate). These complexes were evaluated for their cytotoxic effects against a panel of cancer cell lines (Caco-2, HepG2, and MCF-7) and a noncancerous cell line (Vero). Cell viability assays and morphological analysis revealed promising cytotoxic activities for all three complexes against certain cancer cell lines. The observed cell morphology changes in cancer cells, such as cell shrinkage and membrane blabbing, suggested the induction of apoptosis. While varying degrees of selectivity were observed across the complexes and cell lines, these findings demonstrate the potential of naphthyl-substituted ruthenium(II) complexes as a promising class of anticancer agents. The complexes exhibited varying degrees of cytotoxicity, with some demonstrating promising activity against specific cancer cell lines. Studies on DNA and BSA binding, as well as molecular docking simulations, provided insights into potential mechanisms of action, such as DNA intercalation and protein binding. Their ability to selectively target cancer cells and induce apoptosis is encouraging. Furthermore, Hirshfeld surface analysis was employed to gain a deeper understanding of intermolecular interactions within the crystal structures of the complexes. These analyses provided valuable information on the nature and strength of intermolecular forces, which can influence the physicochemical properties and biological activity of the compounds. Future research will explore these findings in more detail, including in vivo studies, to assess their therapeutic potential and optimize their selectivity further.

## 4. Experimental

### 4.1. Chemistry

#### 4.1.1. General Information

Reactions that involved air-sensitive components were performed using a Schlenk-type flask under argon atmosphere and high-vacuum line techniques. The glass equipment was heated under a vacuum to remove oxygen and moisture, following which it was filled with argon. Chemicals were purchased from Merck, Sigma, or Alfa Aesar. ^1^H, ^13^C, ^19^F, ^31^P, and 2D NMR spectra were recorded on a Varian 400 MHz spectrometer. RuCl_2_(*p*-cymene)]_2_ was prepared according to the method reported by Bennett and Smith through the reaction of Ru(III) chloride with *α*-terpinene [45]. All complexes were checked for purity before the biological testing.

#### 4.1.2. General Procedure for the Synthesis of **Ru1**

N1-(naphthalen-1-yl)ethane-1,2-diamine hydrochloride (1.00 g, 4.50 mmol) and (RuCl_2_[*p*-cymene])_2_ (1.37 g, 2.25 mmol) were dissolved in 20-mL dry MeCN. The mixture was refluxed overnight. NH_4_PF_6_ (0.73 g, 4.50 mmol) was dissolved with MeCN and added the mixture. The mixture was filtered. The filtrate was evaporated to dryness. The crude solid product was purified with column chromatography. Yield: 1.84 g (68%), yellow powder, m.p.: 221°C–223°C. Anal. Calcd. for C_29_H_44_ClF_6_N_2_PRu (702.16): C, 49.61; H, 6.32; N, 3.99. Found: C, 49.59; H, 6.29; N, 4.00. ^1^H NMR (400 MHz, DMSO-d_6_): 8.66 (m, 1 H, Ar-H), 8.07 (m, 1 H, Ar-H), 7.91 (m, 1 H, Ar-H), 7.67 (m, 4 H, Ar-H), 6.52 (br, 1 H, NH), 6.37 (br, 1 H, NH), 5.06 (d, *J* = 5.6 Hz, 1 H, *p*-cymene-Ar-H), 4.97 (d, *J* = 6.0 Hz, 1 H, *p*-cymene-Ar-H), 4.90 (br, 1 H, NH), 4.59 (d, *J* = 5.6 Hz, 1 H, *p*-cymene-Ar-H), 4.34 (d, *J* = 5.6 Hz, 1 H, *p*-cymene-Ar-H), 3.26 (m, 2 H, CH_2_), 2.88 (m, 2 H, CH_2_), 2.43 (m, 1 H, *p*-cymene-CH), 1.78 (s, 3 H, *p*-cymene-CH_3_), 1.04 (d, *J* = 6.8 Hz, 3 H, *p*-cymene-CH_3_), 1.01 (d, *J* = 6.8 Hz, 3 H, *p*-cymene-CH_3_). ^13^C NMR (100 MHz, DMSO-d_6_): 146.53, 134.02, 128.97, 127.25, 126.46, 126.29, 125.88, 125.77, 124.02, 115.23, 106.95, 94.31, 86.05, 83.41, 79.66, 77.51, 53.95, 41.32, 30.20, 22.28, 22.00, 17.20. ^19^F NMR (376 MHz, DMSO-d_6_): −71.12, −69.23 (d, *J* = 711.01 Hz, PF_6_). ^31^P NMR (161 MHz, DMSO-d_6_): −144.22 (septet, *J* = 706.8 Hz, PF_6_).

#### 4.1.3. General Procedure for the Synthesis of **Ru2**

**L2** (0.50 mg, 1.47 mmol) and triethylamine (0.15 mg, 1.47 mmol) were stirred in 20 mL DCM at RT for 1 h, and then, (RuCl_2_(*p*-cymene])_2_ (0.45 mg, 0.73 mmol) was added. After the reaction was stirred at RT for overnight, the solvent was evaporated in a vacuum and purification was carried out by column chromatography with DCM. Yield: 520 mg (57%). Anal. Calcd. for C_29_H_33_ClN_2_O_2_RuS (610.17): C, 57.08; H, 5.45; N, 4.59; S, 5.26. Found: C, 57.11; H, 5.42; N, 4.57; S, 5.24. ^1^H NMR (400 MHz, CDCl_3_): 8.86 (d, *J* = 8.0 Hz, 1 H, Ar-H), 7.97 (d, *J* = 8.0 Hz, 1 H, Ar-H), 7.81 (m, 3 H, Ar-H), 7.69 (m, 3 H, Ar-H), 7.43 (d, *J* = 7.6 Hz, 1 H, Ar-H), 7.22 (d, *J* = 8.0 Hz, 2 H, Ar-H), 5.81 (br, 1 H, *p*-cymene-Ar-H), 5.58 (br, 1 H, *p*-cymene-Ar-H), 4.66 (br, 1 H, *p*-cymene-Ar-H), 4.33 (br, 1 H, *p*-cymene-Ar-H), 3.37 (m, 2 H, CH_2_), 2.87 (m, 2 H, CH_2_), 2.57 (m, 1 H, *p*-cymene-CH), 2.38 (s, 3 H, CH_3_), 2.00 (s, 3 H, *p*-cymene-CH_3_), 1.25 (d, *J* = 6.8 Hz, 3 H, *p*-cymene-(CH_3_)_2_), 1.19 (d, *J* = 6.8 Hz, 3 H, *p*-cymene-(CH_3_)_2_). ^13^C NMR (100 Hz, CDCl_3_): 145.76, 140.46, 140.23, 134.01, 128.91, 128.58, 127.50, 127.11, 126.97, 126.43, 125.41, 124.96, 122.67, 114.23, 54.23, 47.73, 30.05, 22.45, 22.19, 21.41, 18.32.

#### 4.1.4. General Procedure for the Synthesis of **Ru3**

Thionalide (0.10 g, 0.46 mmol) was dissolved in 5 mL of dry acetonitrile. RuCl_2_(*p*-cymene)]_2_ (0.14 g, 0.23 mmol) was added to the reaction. The final mixture was stirred overnight at room temperature. Upon filtering the mixture, the filtrate was evaporated to dryness. The crude solid product was purified via column chromatography (dichloromethane/methanol). The precipitate was recrystallized in DCM/Et_2_O. Yield: 0.20 g (32%), brown crystal, m.p.: 252°C. Anal. Calcd. for C_44_H_46_N_2_O_2_Ru_2_S_2_ (901,12): C, 58.65; H, 5.15; N, 3.11; S, 7.12. Found: C, 58.61; H, 5.12; N, 3.09; S, 7.10. ^1^H NMR (400 MHz, CDCl_3_): 7.96 (s, 1 H, Ar-H), 7.84 (m, 3 H, Ar-H), 7.65 (m, 1 H, Ar-H), 7.45 (m, 2 H, Ar-H), 5.31 (d, *J* = 6.0, 1 H, *p*-cymene-Ar-H), 5.19 (d, *J* = 6.0, 1 H, *p*-cymene-Ar-H), 4.95 (d, *J* = 6.0, 1 H, *p*-cymene-Ar-H), 4.89 (d, *J* = 6.0, 1 H, *p*-cymene-Ar-H), 3.57 (q, *J* = 15.6–46.0, 2 H, CH_2_), 2.13 (m, 1 H, *p*-cymene-CH), 1.92 (s, 3 H, *p*-cymene-CH_3_), 1.06 (d, *J* = 6.8, 3 H, *p*-cymene-CH_3_), 0.86 (d, *J* = 6.8, 3 H, *p*-cymene-CH_3_). ^13^C NMR (100 MHz, CDCl_3_): 178.41, 152.04, 134.29, 130.95, 127.99, 127.52, 127.47, 125.62, 124.56, 123.49, 107.62, 99.49, 85.57, 84.68, 83.86, 83.51, 44.43, 30.25, 23.29, 21.62, 17.93.

### 4.2. Pharmacology

#### 4.2.1. MTT (3-(4,5-Dimethylthiazol-2-yl)-2,5-Diphenyl-2H-Tetrazolium Bromide) Assay

The MTT assay, which is based on the reduction of 3-(4,5-dimethylthiazol-2-yl)-2,5-diphenyltetrazolium bromide to a purple formazan product, was performed to estimate cell viability and growth. To determine the appropriate dose and time, we performed the MTT assay in HepG2, MCF-7, Caco-2, and Vero cell lines. Cell suspensions of both cell lines were first prepared at densities of 1 × 10^4^/mL cells per well of 96-well culture dishes and plated in triplicate. Cells were then incubated with the complexes (2.5, 5, 10, 25, and 50 μM) for 24 and 48 h. After the treatment using the complexes, a 10 μL MTT (Glentham Life Sciences, GC4568) solution was added to each well, followed by incubation for 4 h at 37°C in 5% CO_2_. The medium was then discarded, and 50 μL DMSO was added to each well to dissolve the formazan crystals. The absorbance was immediately measured at 540 nm using a UV-visible spectrophotometer multiplate reader.

#### 4.2.2. FS-DNA Binding Studies

UV-visible spectroscopy was employed to investigate the interaction between BSA and **Ru1-3**. Different concentrations of **Ru1-3** (0–15 μM) were titrated against a fixed concentration of FS-DNA (0.1 μM) to monitor changes in absorbance at 260 nm. The analysis of these titration data allowed for the determination of the binding constant (*K*_*b*_) using the following equation:

(1) $$\begin{array}{c} \frac{1}{A-A_{0}}=\frac{1}{A_{\max}-A_{0}}+\frac{1}{\left[C\right]}\times \frac{1}{K_{b}\left(A_{\max}-A_{0}\right)}, \end{array}$$

where *A* is the absorbance of FS-DNA at a specific concentration with increasing complex concentration, *A*_0_ is the absorbance of FS-DNA without complex, *A*_max_ is the absorbance of the FS-DNA complex at the final state, and C is the complex concentration.

#### 4.2.3. BSA Binding Studies

UV-visible spectroscopy was employed to investigate the interaction between BSA and **Ru3**. Different concentrations of **Ru3** (0–15 μM) were titrated against a fixed concentration of BSA (0.1 μM) to monitor changes in absorbance at 290 nm, a wavelength characteristic of aromatic amino acids in BSA. The analysis of these titration data allowed for the determination of the binding constant (*K*_*b*_) using the following equation:

(2) $$\begin{array}{c} \frac{1}{A-A_{0}}=\frac{1}{A_{\max}-A_{0}}+\frac{1}{\left[C\right]}\times \frac{1}{K_{b}\left(A_{\max}-A_{0}\right)}, \end{array}$$

where *A* is the absorbance of BSA at a specific concentration with increasing complex concentration, *A*_0_ is the absorbance of BSA without complex, *A*_max_ is the absorbance of the BSA-complex at the final state, and *C* is the complex concentration.

#### 4.2.4. Molecular Docking Analysis

In this study, molecular docking of the **Ru3** compound was conducted to target BSA, EGFRWT, and the mutant form EGFRT790M. The docking simulations were performed using AutoDock Vina (version 4.2.5.1). Protein structures, including BSA (PDB ID: 3F5S), EGFRWT (PDB ID: 4HJ0), and EGFRT790M (PDB ID: 3W20), were sourced from the RCSB Protein Data Bank (https://www.rcsb.org/) in PDB format. Protein preparation was carried out using the Protein Preparation Wizard, which involved assigning bond orders, introducing hydrogen atoms, processing any metal ions, and eliminating water molecules. Energy minimization was performed until the root mean square deviation (RMSD) reached 0.30 Å. The 3D structures of the ligands were generated through Maestro 8.5 from the Schrödinger suite, while Open Babel software was utilized for generating 3D conformations of the synthesized compounds. For the docking grid, parameters were adjusted to maintain an RMSD under 2 Å. The center of the grid for proteins was defined at coordinates *X* = 21.41, *Y* = 3.62, and *Z* = 21.94, with grid dimensions of 60 Å × 60 Å × 60 Å. The same configuration was employed across all tested proteins after optimization.

Docking produced various conformations, which were analyzed using Discovery Studio to examine the molecular secondary structures. EB was selected as the reference compound for comparison. The chemical structure of **Ru3** was illustrated and optimized using Gaussian 09W, with the optimized form saved as an SDF file and imported into the Maestro graphical user interface (GUI). Ligand preparation was completed using the LigPrep module with standard settings, applying the OPLS 2005 force field at physiological pH.

Subsequently, all ligand conformations were docked within a receptor grid radius of 20 Å. The co-crystallized ligands were used to pinpoint the binding site. They were then redocked to confirm the accuracy of the docking procedure, resulting in RMSD values of less than 2 Å, demonstrating the protocol's reliability.

#### 4.2.5. Hirshfeld Surface Analysis

Hirshfeld surface analysis was performed using CrystalExplorer 21 to investigate intermolecular interactions within the crystal structures of the synthesized ruthenium complexes [46]. The dnorm surface, shape index, and curvedness surfaces were generated to visualize and quantify various aspects of intermolecular contacts, including hydrogen bonding, van der Waals interactions, and molecular shape. Two-dimensional fingerprint plots were generated to provide a quantitative analysis of the types and frequencies of intermolecular contacts [47].

## Figures and Tables

**Figure 13:**
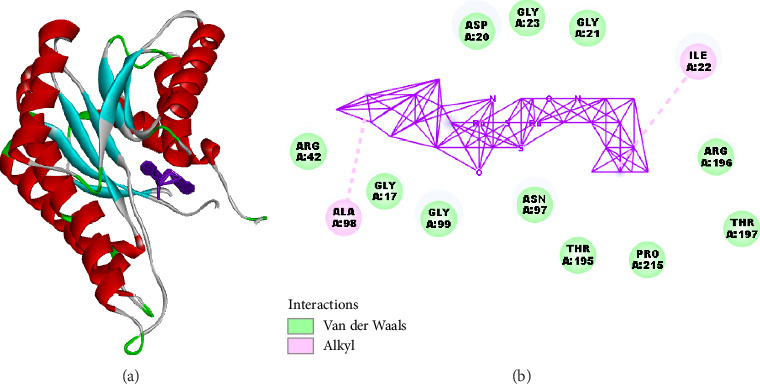
Molecular interaction analysis of the synthesized compound **Ru3** with bovine serum albumin (BSA). (a) 3D structure of BSA with bound **Ru3** compound: This panel displays the 3D structure of BSA, highlighting its secondary structural elements (Figure 13). The red helices represent alpha-helices, which dominate the protein's structure, while the cyan arrows indicate beta-sheets. The loop regions are colored gray. The purple-highlighted region represents the active site where the synthesized compound binds. This visualization illustrates the overall conformation of BSA, showing the complex folding pattern typical of serum albumins, which play a significant role in drug binding due to their abundant alpha-helices and flexible loops. (b) Interaction map of the synthesized compound with BSA active site residues: This panel shows a 2D interaction map of the synthesized compound docked into the active site of BSA (Figure 13). Key interactions between the compound and the amino acid residues are represented, with green indicating van der Waals interactions and pink representing alkyl interactions. Van der Waals interactions are observed with residues ASP A:20, GLY A:17, GLY A:21, GLY A:23, ASN A:97, GLY A:99, THR A:195, PRO A:215, ARG A:196, and THR A:197. These interactions suggest that the synthesized compound is stabilized within the hydrophobic pocket of BSA through noncovalent, weak attractive forces. Alkyl interactions are noted with residues ALA A:98 and ILE A:22, indicating hydrophobic interactions where the alkyl chains of the compound interact with nonpolar regions of the BSA protein.

**Figure 14:**
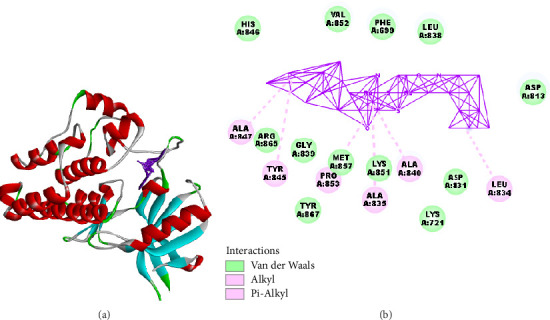
Molecular interaction analysis of the synthesized compound **Ru3** with EGFR wild type (EGFRWT). (a) 3D structure of EGFRWT with bound **Ru3** compound: This panel displays the 3D structure of the EGFR wild type (EGFRWT) protein, showcasing the secondary structure elements (Figure 14). Red helices represent alpha-helices, while cyan arrows indicate beta-sheets. The loop regions are depicted in gray, highlighting the flexible parts of the protein. The binding pocket, where the synthesized compound **Ru3** interacts, is indicated by a purple-colored region. The figure illustrates the spatial arrangement of the active site and the overall conformational framework of EGFRWT, providing a visual representation of the docking site for **Ru3**. (b) Interaction map of **Ru3** with key residues of EGFRWT active site: This panel presents a detailed 2D interaction map showing the molecular interactions between **Ru3** and key amino acid residues within the active site of EGFRWT (Figure 14). van der Waals interactions (marked in green) are observed with residues such as VAL A:852, PHE A:699, LEU A:838, HIS A:846, GLY A:839, PRO A:853, MET A:857, ASP A:813, and LYS A:721. These weak, noncovalent forces help stabilize the compound within the binding pocket, enhancing its interaction with the target protein. Alkyl interactions (highlighted in pink) are identified with residues ALA A:835, ALA A:840, ALA A:847, LEU A:834, and MET A:857. These hydrophobic contacts occur between the alkyl chains of the **Ru3** compound and nonpolar regions of the EGFRWT protein, which contribute to the binding affinity. Pi-Alkyl interactions are depicted with residues TYR A:845, ARG A:865, and TYR A:867, suggesting that aromatic rings of **Ru3** interact with the alkyl groups of the protein residues, enhancing stability through *π*-alkyl stacking.

**Figure 15:**
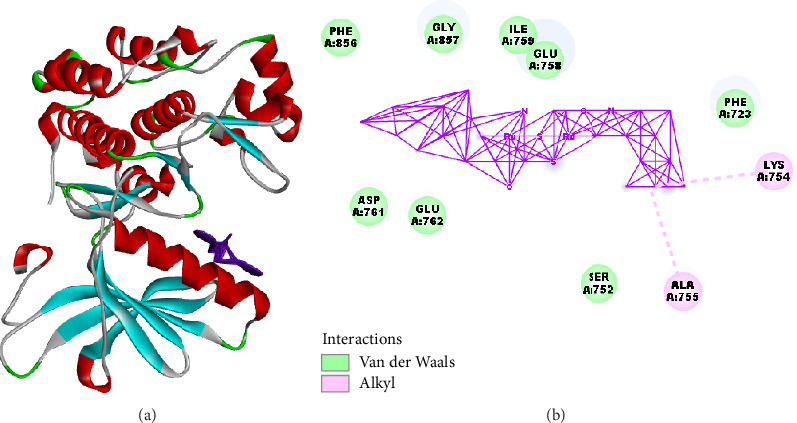
Molecular interaction analysis of the synthesized compound **Ru3** with mutant EGFR (EGFR T790M). (a) The 3D structural model of mutant EGFRT790M bound to the synthesized compound **Ru3**. The protein structure is displayed as a ribbon diagram, with different secondary structures represented in various colors: helices in red, beta-sheets in cyan, and loops in gray (Figure 15). The green segments highlight areas involved in van der Waals interactions, while the purple structure in the binding pocket represents the compound **Ru3**. This view provides an overview of the binding orientation of **Ru3** within the EGFR T790M mutant's active site, emphasizing potential interaction zones. (b) Interaction map of **Ru3** with surrounding amino acid residues in EGFR T790M. Each labeled circle represents a specific amino acid residue involved in interactions with **Ru3** (Figure 15). The green circles indicate residues involved in van der Waals interactions, while the pink circles denote residues engaged in alkyl interactions. Solid purple lines represent van der Waals contacts between **Ru3** and the surrounding amino acids, forming a dense interaction network within the binding pocket. A dashed pink line shows an additional alkyl interaction, contributing to the stability of **Ru3** binding within the mutant EGFR pocket. Residues such as LYS A:754 and ALA A:755 are highlighted for their key roles in stabilizing the interaction through alkyl contacts, while residues like PHE A:856, GLY A:857, and ILE A:759 form van der Waals interactions.

**Table 1:** IC_50_ values of Ru complexes in MCF-7, Caco-2, Vero, and HepG2 cell lines.

	MCF-7 (μM)	Caco-2 (μM)	HepG2 (μM)	Vero (μM)
**Ru1** (24 h)	3.99	9.22	5.50	7.84
**Ru1** (48 h)	11.69	3.83	6.00	9.38
**Ru2** (24 h)	2.81	1.39	4.72	7.80
**Ru2** (48 h)	8.75	3.39	8.80	38.34
**Ru3** (24 h)	5.18	5.88	10.89	5.92
**Ru3** (48 h)	8.09	1.81	10.22	6.55

**Table 2:** Molecular docking analysis of **Ru3** and reference drug EB with BSA, EGFRWT, and EGFRT790M proteins (EB: Erlotinib).

	BSA			EGFRWT			EGFRT790M		
	Docking score (kcal/mol)	H bond interaction	Other interaction	Docking score (kcal/mol)	H bond interaction	Other interaction	Docking score (kcal/mol)	H bond interaction	Other interaction
**Ru3**	−178.827	−3.110	ASP20, GLY17, GLY99, GLY21, GLY23, ILE22, ARG196, THR195, THR197, PRO215, ASN97, ALA98, ARG42	−204.437	—	HIS846, VAL852, PHE699, LEU834, LEU838, ASP813, ASP831, LYS721, LYS851, ALA835, ALA840, ALA847, MET857, PRO853, TYR845, TYR867, GLY839, ARG865	−176.946	—	PHE856, GLY857, ILE759, GLU758, GLU762, PHE723, LYS754, ALA755, SER752, ASP761
EB	−153.346	−13.107	ARG42, ARG196, THR195, THR148, PRO192, LEU40, LEU47, LEU100, LEU101, ASN43, ASN122, ILE121, TYR163, GLY41, GLY99, ALA18, ALA98, ER19, LYS46	−179.941	−4.057	PHE699, LEU838, ALA835, ALA840.	−150.700	−0.598	MET790, MET766, LEU844, LEU718, LEU792

## Data Availability

All data supporting the results are included within the article and in the Supporting Information.

## Supporting Information

The supporting information file contains detailed NMR analysis results of the complexes (Figure S1–S8), DNA and BSA binding graphs (Figure S9–S12), and microscope images of cell morphology (Figure S13–S16).
