# Supplementary material for: Naphthyl-Substituted Ruthenium(II)–Arene Complexes: Exploring the Impact of Binding Modes on Cytotoxicity in Cancer and Normal Cell Lines
Source: Bioinorg Chem Appl. 2025 May 4;2025:5556956. doi: 10.1155/bca/5556956 (PMC12066179; doi:10.1155/bca/5556956)
Supplement: Supporting Information — Additional supporting information can be found online in the Supporting Information section. [file 5556956.f1.docx]

**Supporting Information**

**Naphthyl-Substituted Ruthenium(II) Arene Complexes: Exploring the Impact of Binding Modes on Cytotoxicity in Cancer and Normal Cell Lines**

**Serdar Batıkan Kavukcu^1^, Hafize Seda Vatansever^2,3^, Süleyman İlhan,^4^ Hayati Türkmen^1*^**

^1^ Department of Chemistry, Faculty of Science, Ege University, Izmir, Türkiye.

^2^ Department of Histology and Embryology, Faculty of Medicine, Manisa Celal Bayar University, Manisa, Türkiye.

^3^ DESAM Institute, Near East University, Mersin 10, Türkiye.

^4^ Department of Biology, Faculty of Engineering and Natural Sciences, Manisa Celal Bayar University, Manisa, Türkiye.

**Figure S1.** ^1^H and ^13^C NMR spectra of **Ru1** (DMSO-*d_6_*).

**Figure S2.** ^19^F and ^31^P NMR spectra of **Ru1** (DMSO-*d_6_*).

**Figure S3.** 2D ^1^H-^1^H gCOSY and ^1^H-^13^C gHSQC NMR spectra of **Ru1** (DMSO-*d_6_*).

**Figure S4.** 2D 1H-1H gHMBC NMR spectrum of **Ru1** (DMSO-*d_6_*).

**Figure S5.** ^1^H and ^13^C NMR spectra of **Ru2** (CDCl_3_).

**Figure S6.** ^1^H and ^13^C NMR spectra of **Ru3** (CDCl_3_).

**Figure S7.** 2D ^1^H-^1^H gCOSY and ^1^H-^13^C gHSQC NMR spectra of **Ru3** (CDCl_3_).

**Figure S8.** 2D 1H-1H gHMBC and 1H-13C gNOSY NMR spectra of **Ru3** (CDCl_3_).


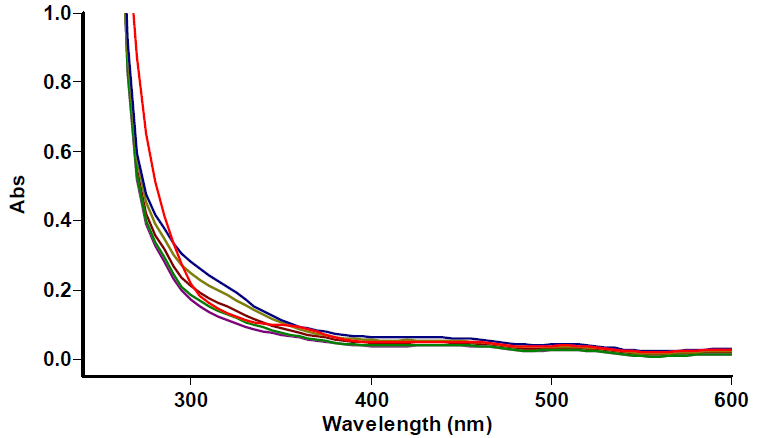


**Figure S9.** Absorption spectra of FS-DNA with increasing concentrations of **Ru1**.


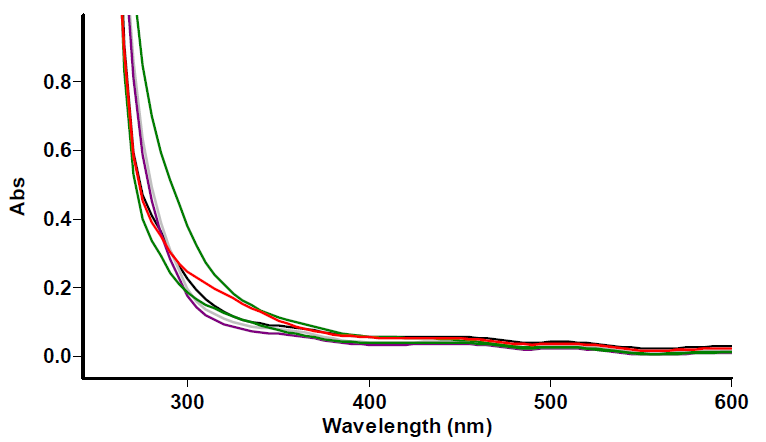


**Figure S10.** Absorption spectra of FS-DNA with increasing concentrations of **Ru2**.


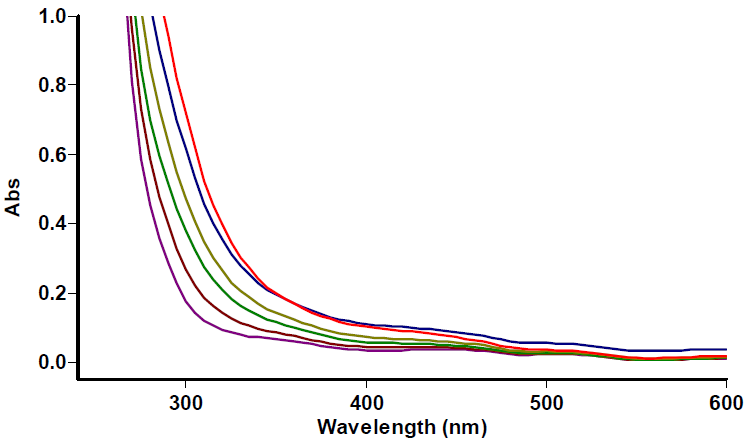


**Figure S11.** Absorption spectra of FS-DNA with increasing concentrations of **Ru3**.


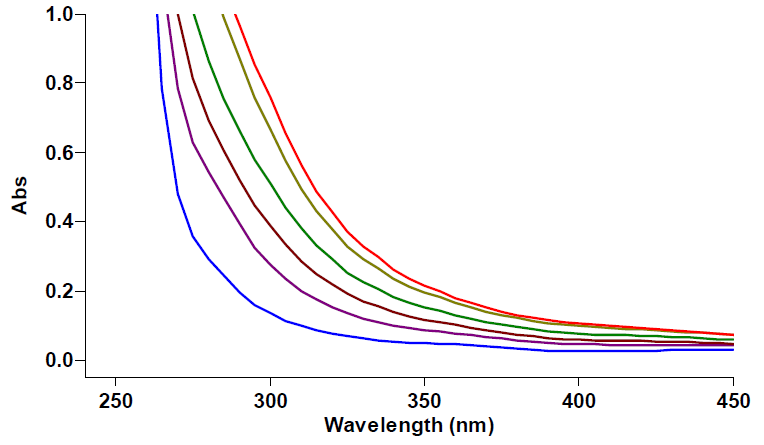


**Figure S12.** UV-Vis spectra of 0.1 µM BSA in Tris-HCl buffer with increasing concentration of **Ru3** (0-15 µM). Inset: 1/(A-A_0_) versus 1/[complex]


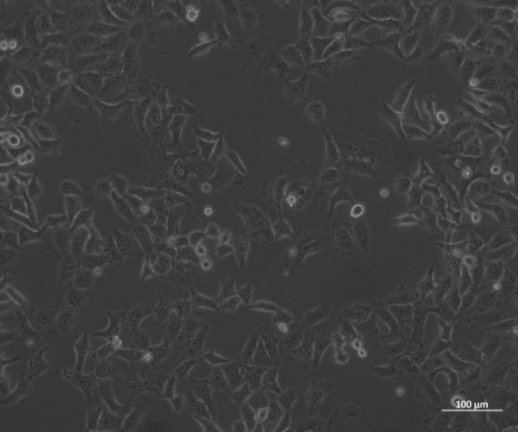

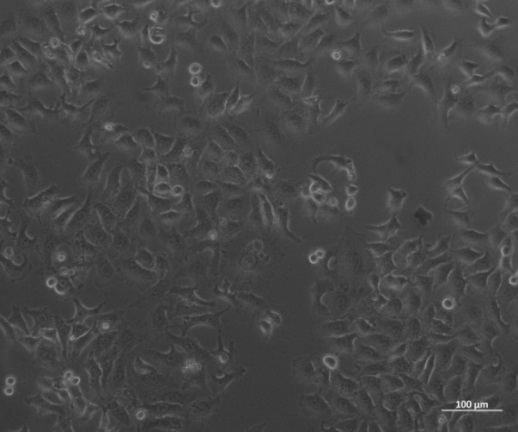


**A**

B


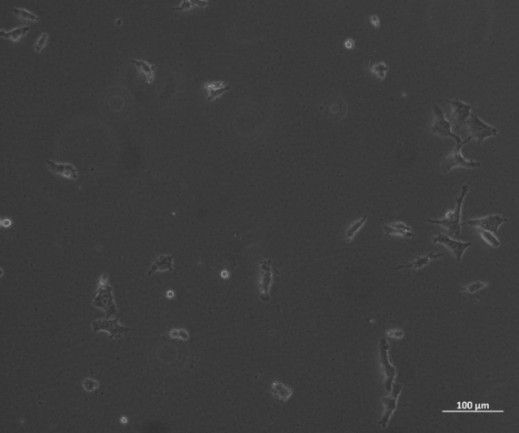

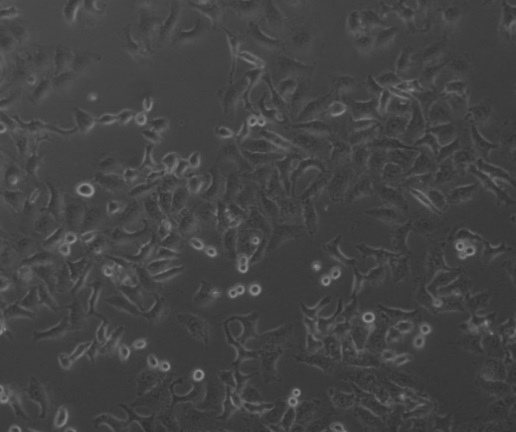


D

C

**Figure S13.** Control (A), 25 μM of **Ru1** treatment (B), **Ru2** μM treatment (C) and 24 h after **Ru3** μM treatment (D) images of MCF7 cell cultures. Scale: 100 μm.


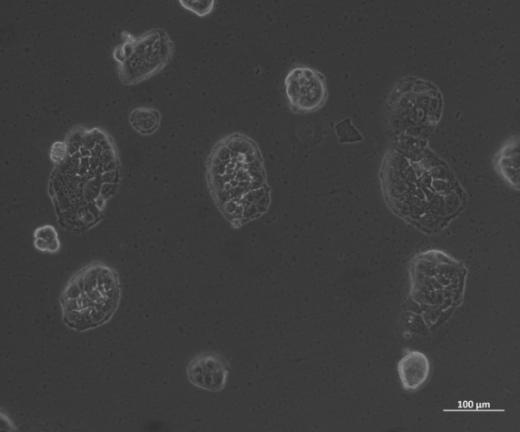

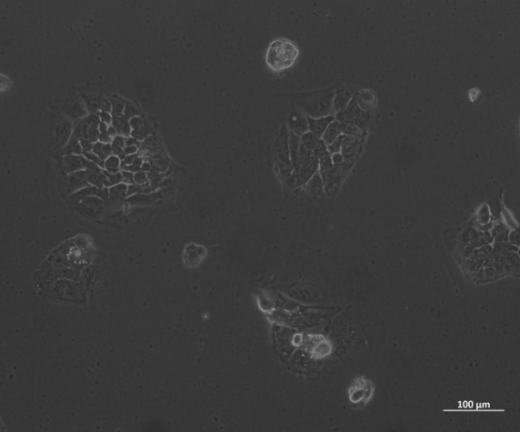


**A**

B


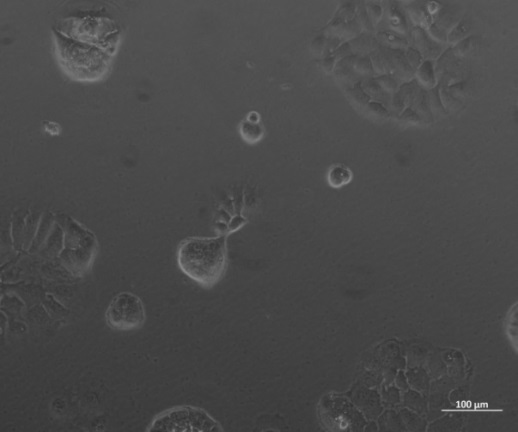

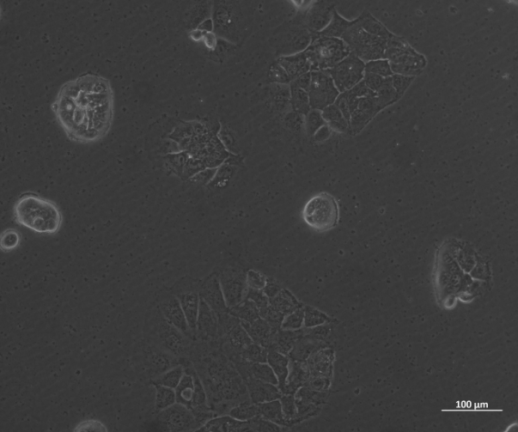


D

C

**Figure S14.** Control (A), 25 μM of **Ru1** treatment (B), **Ru2** μM treatment (C) and 24 h after **Ru3** μM treatment (D) images of Caco-2 cell cultures. Scale: 100 μm.


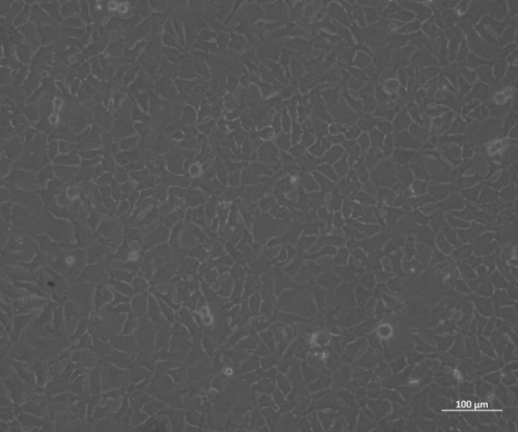

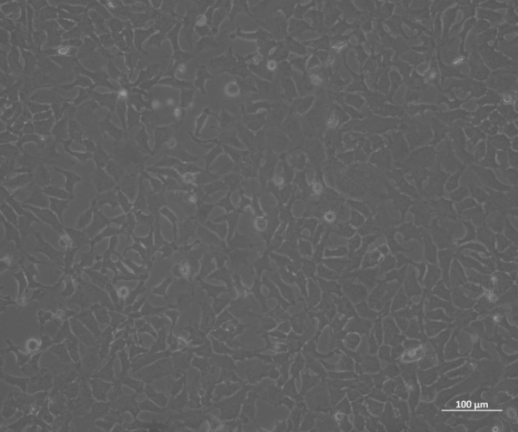


B

C

**A**


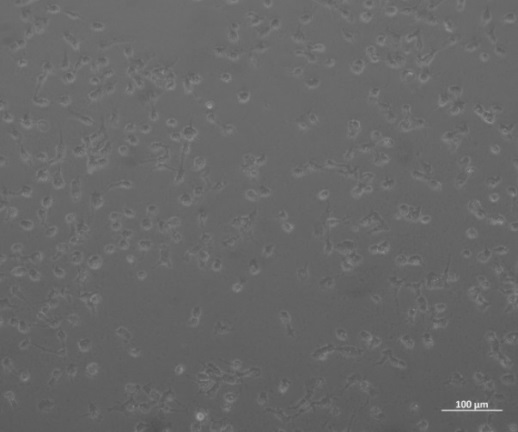

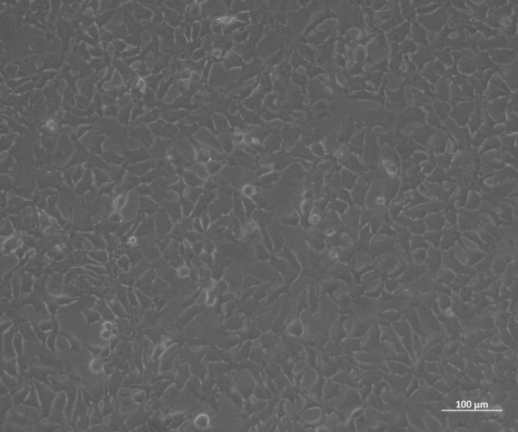


D

**Figure S15.** Control (A), 25 μM of **Ru1** treatment (B), **Ru2** μM treatment (C) and 24 h after **Ru3** μM treatment (D) images of HepG2 cell cultures. Scale: 100 μm.


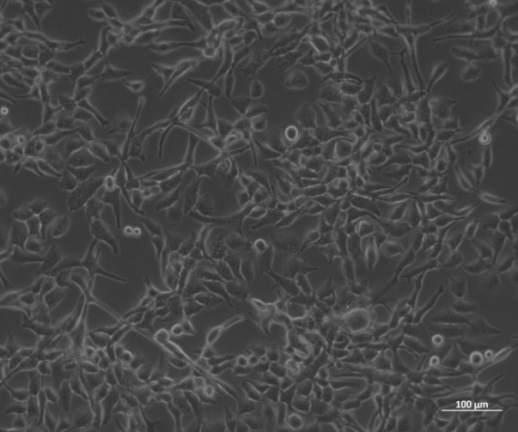

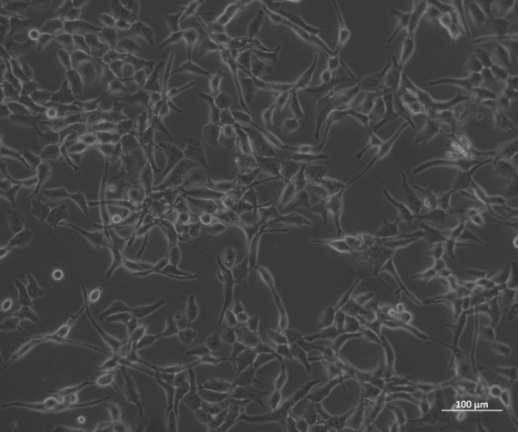


B

**A**


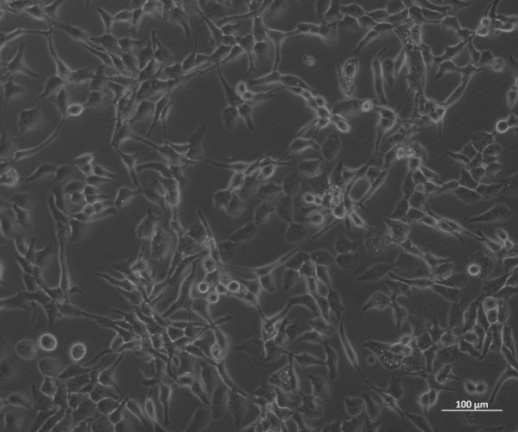

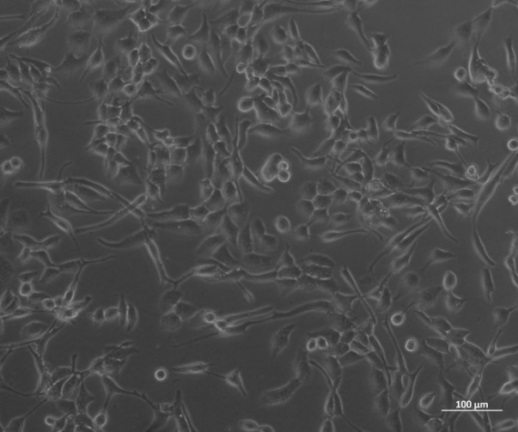


D

C

**Figure S16.** Control (A), 25 μM of **Ru1** treatment (B), **Ru2** μM treatment (C) and 24 h after **Ru3** μM treatment (D) images of Vero cell cultures. Scale: 100 μm.
